# Prognostic significance of tumor-infiltrating immune cells and PD-L1 expression in esophageal squamous cell carcinoma

**DOI:** 10.18632/oncotarget.15621

**Published:** 2017-02-22

**Authors:** Yubo Jiang, Anthony W.I. Lo, Angela Wong, Wenfeng Chen, Yan Wang, Li Lin, Jianming Xu

**Affiliations:** ^1^ Department of Gastrointestinal Oncology, Affiliated Hospital Cancer Center, Academy of Military Medical Sciences, Beijing, P. R. China; ^2^ Division of Anatomical Pathology, Department of Pathology & Clinical Biochemistry, Queen Mary Hospital, Hong Kong Special Administrative Region, P. R. China; ^3^ Global Early Development, Merck Serono China, Beijing, P. R. China

**Keywords:** esophageal squamous cell carcinoma, PD-L1, TILs, prognostic factor, tumor microenvironment

## Abstract

Programmed death-1 receptor (PD-1) and its ligand (PD-L1) play an integral role in regulating the immune response against cancer. This study investigated the prognostic significance of PD-L1 expression on tumor cells and tumor-infiltrating immune cells (TILs) in the tumor microenvironment in Chinese patients with esophageal squamous cell carcinoma (ESCC). Archival formalin-fixed, paraffin-embedded ESCC samples from treatment-naïve patients with ESCC after surgery or by diagnostic endoscopic biopsy were collected between 2004 and 2014. Expression of PD-L1 in ESCC tumor specimens was assessed by immunohistochemistry (IHC), and the degree of TIL infiltration was evaluated by examining hematoxylin and eosin-stained (H&E) specimens. PD-L1+ as defined as ≥1% of tumor cell membranes showing ≥1+ intensity. In 428 patients, specimens from 341 (79.7%) were PD-L1+. In the definitive treatment group (patients who received curative esophagectomy or definitive [chemo-]radiation therapy), PD-L1 positivity was associated with a significantly shorter DFS and OS. In the palliative chemotherapy group exhibited, neither PFS nor OS correlated significantly with PD-L1 expression. PD-L1 expression was positively associated with TIL density. In 17 paired tumor tissues collected before and after treatment, an increase in PD-L1 expression was associated with disease progression, whereas a decrease in PD-L1 expression was associated with response to chemotherapy or disease control. So, PD-L1 expression was associated with a significantly worse prognosis in patients with ESCC. These observations suggest that PD-L1 may play a critical role in ESCC cancer progression and provide a rationale for developing PD-L1 inhibitors for treatment of a subset of ESCC patients.

## INTRODUCTION

Esophageal cancer is one of the most aggressive and lethal malignancies among gastrointestinal cancers, ranking sixth in cancer-related death. It has two main subtypes: squamous-cell carcinoma (ESCC), which is mostly found in Asia, Africa, and South America, and adenocarcinoma (EAC), which is mostly found in North America and Europe [1]. In China, ESCC accounts for more than 90% of cases of esophageal cancer. Surgical resection remains the primary curative treatment and is the priority for localized and locally advanced esophageal cancer. Unfortunately, despite using neoadjuvant therapy followed by complete resections, long-term outcomes remain poor due to high rates of locoregional and distant failure [2, 3]. For patients with unresectable ESCC or who are medically unfit for surgery, definitive (chemo-)radiation may be a good choice, but local and distant recurrence still considerably affect prognosis [4]. Systemic palliative chemotherapy is the main treatment option for metastatic ESCC. However, objective response rates, especially complete response rates, are still unsatisfactory [5, 6]. The most commonly used chemotherapy regimen is cisplatin combined with 5-fluorouracil (5-FU) or/and taxanes, after which there is no standard treatment option [7]. Therefore, there is a crucial need to seek a new direction for treatment.

Programmed cell death 1 (PD-1), a member of the CD28 family of co-stimulatory receptors, is a T cell immune-checkpoint involved in providing inhibitory signals in T-cell activation when engaged by its ligands: PD1 ligand 1 (PD-L1) and PD1 ligand 2 (PD-L2) [8]. The PD-1/PD-L pathway plays a critical role in regulating the activity of T cells in effector phases against tumor cells [9]. It is well established that multiple solid tumor types, including melanoma, renal cell carcinoma (RCC), non-small cell lung cancer (NSCLC), thymoma, ovarian, and colorectal cancer, generate an immunosuppressive tumor microenvironment and avoid T cell-mediated cytolysis by expressing PD-L1 [10–12]. Antibodies blocking PD-1/PD-L1 pathway have demonstrated durable responses in different advanced malignancies. To date, four immune checkpoint inhibitor antibodies have been approved by the U.S. FDA. These include the cytotoxic T-lymphocyte-associated protein 4 (CTLA-4) blocking antibody ipilimumab (for advanced melanoma), two antibodies blocking PD-1, pembrolizumab (for advanced melanoma, NSCLC, and head and neck cancer) and nivolumab (for advanced melanoma, NSCLC, RCC and Hodgkin lymphoma), and one antibody blocking PD-L1, atezolizumab (for advanced urothelial carcinoma). All of these antibodies are thought to mediate their antitumor activity by blocking CTLA-4 or PD-1 on effector immune cells (such as CD8+ T cells) or PD-L1 on the tumor cells. This release of suppression on effector cells thus allows their full antitumor function to be exerted. Additionally, preliminary clinical data suggested that patients with higher levels of PD-L1 expression by immunohistochemistry (IHC) have an improved response rates, PFS, and OS across histologies [13–15]. Thus, PD-L1 expression may be a predictive biomarker for anti-PD-1/PD-L1 therapy [8]. In addition to PD-L1 expression, presence or absence of tumor-infiltrating lymphocytes (TILs) in the tumor microenvironment is also important for effective antitumor immunity. Early research showed that a high degree of CD8+ and CD4+ T-cell infiltration in ESCC correlated with favorable clinical outcome, suggesting that the tumor antigen-specific cellular immune response plays a role in the clinical disease course [16–18]. However, the clinical significance of PD-L1 expression and TILs in patients with ESCC has not been fully investigated in a Chinese patient population.

To explore the prognostic significance of PD-L1 expression and TILs for ESCC, we investigated the extent of PD-L1 expression and TILs in formalin-fixed samples from 428 Chinese ESCC patients, and analyzed the association between PD-L1 expression, TIL density and outcome.

## RESULTS

### Patient demographics and disease characteristics

A total of 428 ESCC specimens, one from each patient, were obtained from patients admitted to the Affiliated 307 Hospital Cancer Center, Beijing, P. R. China during 2004 to 2014. Samples were collected either by surgery (30.60%, n=131) or by endoscopic biopsy (69.40%, n=297) from treatment-naïve patients. The median follow-up was 34.4 months (range 0.3-147.1 months) from the date of sample collection. The last follow-up date was December 31, 2015. At the time of last follow-up, 22.43% (n=96) of patients with ESCC were still alive.

Treatment modalities depended on the stage and localization of ESCC. Patients with localized tumors underwent curative esophagectomy (n=172, 40.18%), while those with unresectable disease or who were medically unfit for surgery received definitive (chemo-)radiation (n=101, 23.60%). Patients with advanced metastatic disease received palliative chemotherapy (n=90, 21.02%), palliative R1/R2 resection (n=9, 2.10%), best supportive care (n=22, 5.14%), or chose not to receive any treatment (n=34, 7.94%).

### PD-L1 expression in ESCC specimens

**Figure 1:**
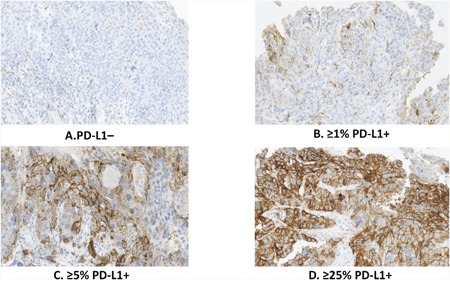
Tumor PD-L1 expressed at different levels in ESCC specimens **A**. PD-L1 negative; **B**. ≥1% PD-L1-positive tumor cells with membrane staining of any intensity; **C**. ≥5% PD-L1-positive tumor cells with membrane staining of any intensity; **D**. ≥25% PD-L1-positive tumor cells with membrane staining of at least 2+ intensity.

Tumor PD-L1 expression was assessed via IHC, using rabbit monoclonal antibody clone 73-10 proprietary of Merck KGaA. Table 1 summarizes the percentage of specimens PD-L1+ using different scoring criteria: ≥1% (Figure 1B) and ≥5% (Figure 1C) of tumor cells showing ≥1+ membrane staining intensity, as well as ≥25% tumor cells showing ≥2+ membrane staining intensity (Figure 1D). Among 428 ESCC specimens, 79.7%, 32.7% and 9.1% were PD-L1+ tumors using cutoff values of 1%, 5% and 25%, respectively. There was no significant difference in percentages of PD-L1+ specimens tumors between those obtained via surgery or biopsy (46/131, 35.1% PD-L1+ in surgical specimens versus 94/297, 31.6% PD-L1+ in biopsy specimens, p=0.481), suggesting that PD-L1 expression level was not affected by size of specimens.

**Table 1:** Distribution of PD-L1+ specimens using different scoring criteria (N=428)

Criteria for positivity	Percentage of PD-L1+ Specimens					
	Tumor cells ≥1%*	Tumor cells ≥5%^@^	Tumor cells ≥25%^#^	Stromal cells ≥1%	Immune cells ≥1%	All cell Types ≥1%
**PD-L1+**	341(79.67)	140(32.71)	39(9.11)	245(57.24)	57(13.32)	407(95.09)
**PD-L1-**	87(20.32)	288(67.29)	389(90.89)	183(42.76)	371(86.68)	21(4.91)

^*^ Primary criteria used in this study: ≥1% expression on tumor cell membranes with ≥1+ intensity;

^@^ ≥5% expression on tumor cell membranes with ≥1+ intensity;

^#^≥25% expression on tumor cell membranes with ≥2+ intensity.

**Figure 2:**
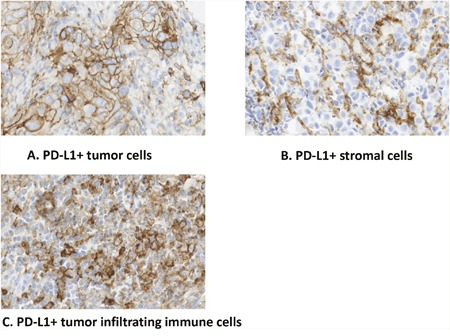
**A**. PD-L1 mainly expresses on the membranes of the tumor cells; **B**. PD-L1 expresses on the membranes of tumor-infiltrating immune cells; **C**. PD-L1 expresses on stromal cells.

PD-L1 expression was found primarily on the membranes of the tumor cells (Figure 2A), and was also expressed on the membranes of tumor-infiltrating immune cells (Figure 2B) and stromal cells (Figure 2C). The rates of PD-L1+ stromal cells and immune cells was 57.2% and 13.3%, respectively (Table 1). When compared to other tumor types, for example, NSCLC, melanoma and nasopharyngeal cancer, ESCC exhibits a lower levels of PD-L1 expression when assayed using the same PD-L1 monoclonal antibody (unpublished data).

**Figure 3:**
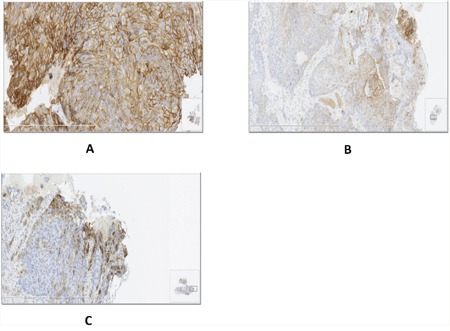
Different patterns of PD-L1 expression in ESCC specimens **A**. Diffuse PD-L1 expression in the presence of TILs; **B**. Regional expression of PD-L1 colocalized with TILs; **C**. PD-L1 expression at the invasive front.

Three major patterns of PD-L1 expression were found in ESCC specimens: (i) a diffuse PD-L1 expression in the presence of TILs; (ii) regional expression of PD-L1 colocalized with TILs; and (iii) PD-L1 expression at the invasive front (Figure 3). PD-L1 expression was positively associated with the amount of TILs (odds ratio=1.837, 95% CI: 1.214, 2.612, p=0.0019) (Table 2).

**Table 2:** PD-L1 expression and TIL density

TIL density score	Patients by PD-L1 expression status*, n (%)		
	Total (n=428)	PD-L1+(n=341)	PD-L1− (n=87)
**None (0)^@^**	27 (6.3)	14 (51.9)	13 (48.1)
**Rare (1)**	131 (30.6)	95 (72.5)	36 (27.5)
**Moderate (2)**	198 (46.3)	174 (87.9)	24 (12.1)
**High (3)**	72 (18.8)	58 (80.6)	14 (19.4)

^*^≥1% of tumor cell membranes with ≥1+ intensity were considered PD-L1+; non-zero correlation, p=0.0019.

^@^ The amount of TILs was evaluated from the H&E stained specimens and assigned a semiquantitative score from 0 to 3, with 0=none, 1=rare, 2=moderate and focal infiltration of tumor, and 3=prominent and diffuse infiltration amongst tumor.

### Correlation of PD-L1 expression and clinicopathological features

Among the 428 patients, the majority 335 (78.3%) were male and 247 (57.7%) were older than 60 years. According to the 7^th^ IUCC/AJCC staging system, 157 (36.7%) and 191 (44.6%) had stage 0-II and III disease, respectively. And the majority (n=255, 59.6%) of tumor specimens showed a moderate to high differentiated tumor. The clinicopathological features of patients are summarized in Table 3.

**Table 3:** Clinicopathologic features of the ESCC patients

Clinicopathologic parameter	No. of patients (%)			
	Total	PD-L1-	PD-L1+	*P^@^*
**Total**	428	87(20.3)	341(79.7)	
**Age(years)**				
≤60	181(42.3)	39(44.8)	142(41.6)	0.591
>60	247(57.7)	48(55.2)	199(58.4)	
**Gender**				
Female	93(21.7%)	19(21.8)	74(21.7)	0.978
Male	335 (78.3%)	68(78.2)	267(78.3)	
**Level of differentiation**				
Moderate to high	257(60.0)	44(50.6)	213(62.5)	0.122
Low	142(33.2)	35(40.2)	107(31.4)	
Others*	29(6.8)	8(9.2)	21(6.2)	
**Primary tumor location^#^**				
Upper thoracic	108(26.9)	17(20.2)	91(28.7)	0.120
Middle and lower thoracic	293(73.1)	67(79.8)	226(71.3)	
**Tumor invasion depth (T) ^#^**				
T0	6(1.8)	4(5.8)	2(0.8)	0.080
T1	24(7.2)	5(7.2)	19(7.2)	
T2	63(18.9)	13(18.8)	50(18.9)	
T3	196(58.9)	40(58.0)	156(59.1)	
T4	44(13.2)	7(10.1)	37(14.0)	
**Nodal metastasis (N) ^#^**				
N0	197(51.6)	49(62.8)	148(48.7)	0.026
N+	185(48.4)	29(37.2)	156(51.3)	
**TNM stage^#^**				
0-II	143(39.9)	33(46.5)	110(38.3)	0.183
III	175(48.9)	34(47.9)	141(49.1)	
IV	40(11.2)	4(5.6)	36(12.5)	

^*^ Others mainly include high-grade dysplasia, carcinoma *in situ*

#According to the 7^th^ IUCC/AJCC staging system, location of the primary cancer site was defined by the position of the upper (proximal) edge of the tumor in the esophagus, and the remaining missing patients were ominous

@The statistical method was chi-square test.

Statistical analyses using Spearman Rank Correlation indicated that PD-L1+ specimens were associated with aggressive clinicopathological features, which included deeper tumor invasion (p=0.037) and nodal metastasis (p=0.013). No significant association was found between PD-L1 expression and gender (p=0.545), age (p=0.943), level of differentiation (p=0.955), primary tumor location (p=0.231) or TNM stage (P=0.581).

### Prognostic significance of PD-L1 expression

The correlation of PD-L1 expression with disease prognosis, as measured by disease free survival (DFS/PFS) and overall survival (OS), was analyzed separately in the definitive treatment cohort and palliative treatment cohorts.

**Figure 4:**
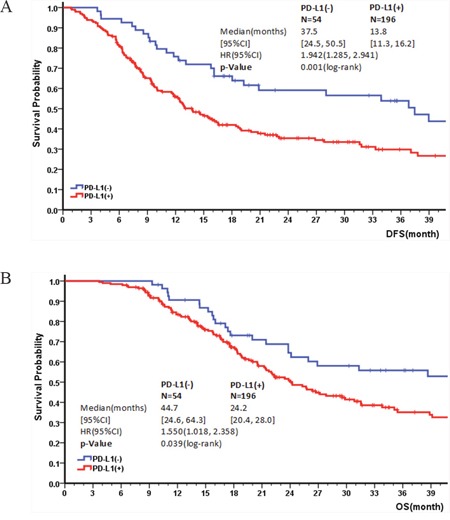
A & B: Correlation of PD-L1 expression and DFS/OS in the definitive treatment cohort

The definitive treatment cohort comprised 250 patients, including patients who received curative esophagectomy or definitive (chemo-)radiation therapy, and excluding those who had hospital mortality (n=16) and occurrence of other primary cancer (n=7). The DFS rates at 1, 3, and 5 years, respectively, were 79.5%, 53.9%, 32.3% for PD-L1– patients and 57.8%, 31.1%, 21.3% for PD-L1+ patients. Similarly, the OS rates at 1, 3, and 5 years, respectively, were 92.5%, 55.8%, 36.5% for PD-L1– patients and 84.5%, 36.3%, 29.8% for PD-L1+ patients. DFS and OS was significantly related to PD-L1 expression (HR=1.942, 95% CI: 1.285, 2.941, p=0.001; HR=1.550, 95% CI: 1.018, 2.358, p=0.0039), PD-L1+ patients exhibited worse DFS and OS than PD-L1– patients (Figure 4A & 4B). Statistical analyses of independent factors associated with DFS and OS in definitive treatment cohort indicated that DFS was significantly related to TNM status (HR=1.541, 95% CI: 1.034, 2.229, p=0.034) and PD-L1 expression (HR=2.336, 95% CI: 1.441, 3.106, p=0.001). Similarly, OS was negatively correlated with PD-L1 expression (HR=1.873, 95% CI: 1.155, 2.331, p=0.01) and TNM status (HR=1.413, 95% CI: 0.879, 2.602, p=0.040).

Patients in the palliative treatment cohort exhibited no significant association between PD-L1 expression and PFS (HR=0.994, 95% CI: 0.404, 2.449, p=0.990) or OS (HR=1.113, 95% CI: 0.598, 2.069, p=0.736) (Supplementary Figure 1A & 1B).

### TIL density and patient prognosis

**Figure 5:**
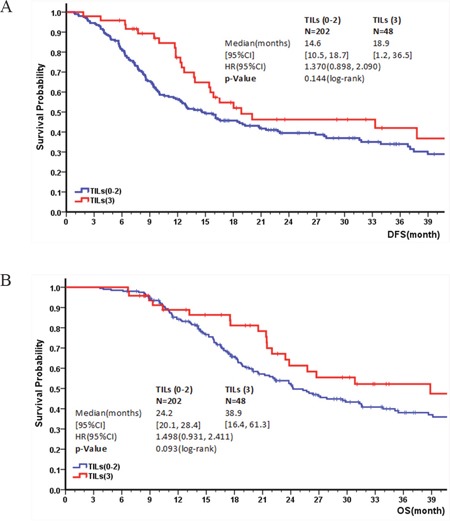
Kaplan–Meier survival curve for DFS and OS depending on TIL level: A & B: Correlation of TIL level and DFS/OS in the definitive treatment cohort

The correlation between TIL density and patient prognosis was examined. Similar to PD-L1 expression, there was no significant difference in TIL density between surgical and biopsy specimens (p=0.243), suggesting that TIL density was not affected by size of the specimens. In definitive treatment cohort, no significant correlation was observed between DFS/OS and TIL density (HR=1.370, 95% CI: 0.898, 2.090, p=0.144; HR=1.498, 95% CI: 0.931, 2.411, p=0.093) (Figure 5A & 5B). However, patients who received esophagectomy who had a high TIL score (3) showed a significantly better OS than those with a moderate (2), low (1) or none (0) TIL score (HR=2.264, 95% CI: 1.039, 4.936, p=0.035). In addition, there was no significant association between PFS or OS with TIL density in the palliative cohort (Supplementary Figure 2A & 2B).

### Changes in PD-L1, CD8 and Foxp3 in paired specimens

Expression of PD-L1 and numbers of CD8+ and Foxp3+ cells was examined in 17 paired tumor specimens. This included 3 patients who did not receive any treatment between the times when paired tumor samples were harvested (mean duration 1.16 ± 0.28 months), 9 paired specimens collected before and after 2 cycles of chemotherapy (mean duration 1.23 ± 0.24 months), and 5 paired specimens collected before and after 4 or more cycles of chemotherapy (mean duration 4.97 ± 2.22 months).

**Figure 6:**
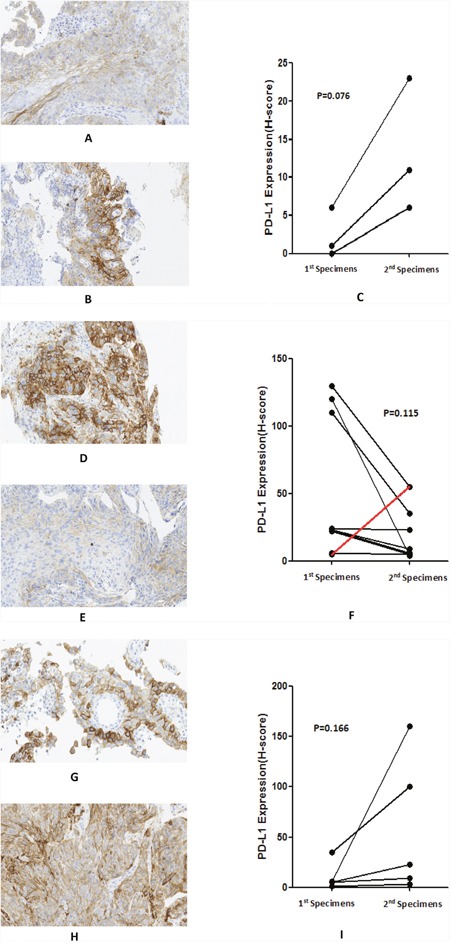
Changes in PD-L1 expression in paired specimens before and after different treatments: A-C: no treatment, H-score from 6 to 23; D-F: 2 cycles chemotherapy (just 1 sample showed an increase [red line]), H-score from 120 to 4; G-I: 4 or more cycles chemotherapy, H-score from 35 to 100

There was an increase in PD-L1 expression as disease progressed (accompanied by an increase in the depth of tumor invasion) in patients who did not receive any treatment (median PD-L1 H-score increased from 1 to 11, p=0.076), with a mean duration of 1.16 months between obtaining harvesting of the first and second tumor specimens. Representative changes in PD-L1 expression are shown in Figure 6A-6C. For patients who received 2 cycles chemotherapy, PD-L1 expression decreased after 2 cycles chemotherapy (median H-score decreased from 23 to 9, p=0.115). Representative changes of PD-L1 are shown in Figure 6D-6F. Of these 9 patients, 6 had a PR and 3 had SD. In contrast, for patients whose samples were collected after 4 or more cycles of chemotherapy, PD-L1 expression increased, consistent with the worsening prognosis of these patients (median H-score increased from 5 to 100, p=0.166). Representative changes of PD-L1 are shown in Figure 6G-6I. Of these 5 patients, 4 patients exhibited PD and 1 exhibited SD at the time of samples was obtained. None of the changes in PD-L1 H-score above represented a significant difference, potentially because of small sample sizes.

**Figure 7:**
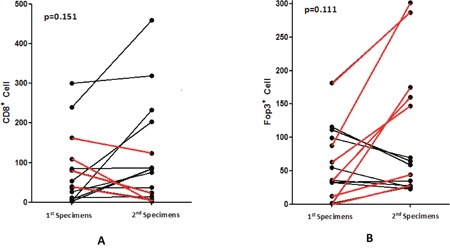
Changes of CD8+ A. and Foxp3+ B. lymphocytes in patients before and after chemotherapy (red line showed the decrease of CD8+ T cells and increase of Foxp3+ T cells)

No statistically significant change was found in CD8+ lymphocyte numbers levels between samples obtained before and after chemotherapy (p=0.151), including patients who received treated with 2 or 4 or more cycles chemotherapy between obtaining harvesting tumor specimens. Of the 14 patients with paired specimens who received chemotherapy, 10 showed an increase of CD8+ lymphocytes while 4 showed a decrease (Figure 7A). Of the 10 samples showing increased CD8+ T cell numbers, there were 6 PR, 3 SD and 1 PD, while of the 4 patients with decreased CD8+ T cell numbers, there were 3 PD and 1 SD. The median OS of the group exhibiting increased CD8+ T cells was higher than that of the group in which CD8+ T cells decreased (12.27 months vs 9.57 months; p=0.157), but the difference was not significant, potentially most likely due to the small sample size. No significance difference was found between samples obtained before and after chemotherapy in Foxp3+ lymphocytes (p=0.111). Among the 14 patients mentioned above, 7 showed an increased in Foxp3+ lymphocytes and other 7 showed a decrease (Figure 7B). Median OS was 12.26 months and 12.30 months respectively (p=0.895).

### Classification based on TILs and PD-L1 expression

Based on PD-L1 status and TIL density, ESCC specimens were classified into 4 groups. In this classification, PD-L1+ was defined as specimens with ≥1% expression on tumor cell membranes with ≥1+ intensity and a TIL score of 3 (high) was considered as TILs+. Type I was PD-L1+ and TILs+ (PD-L1+ with TILs driving adaptive immune resistance), type II was PD-L1– and TILs– (PD-L1–with no TILs indicating immune ignorance), type III was PD-L1+ and TILs– (PD-L1+ with no TILs indicating intrinsic induction), and type IV was PD-L1– and TILs+ (PD-L1– with TILs indicating the role of other suppressor(s) in promoting immune tolerance). The proportions of four types were 13.6% (58/428), 17.1% (73/428), 66.1% (283/428), and 3.3% (14/428), respectively (Table 4). There was a significant difference in OS among four types, with median OS of 30.8, 24.1, 18.3 and 27.0 months respectively for the four types (p=0.007). OS was longer for type II than type III (p=0.034), suggesting that PD-L1 positivity was associated with poor prognosis. Similarly, OS was longer for type 1 than type III (p=0.005), indicating that the presence of TILs were associated with a better prognosis. No significant difference was found between other types.

**Table 4:** Classification of ESCC into 4 groups according to PD-L1 expression and TIL density

	PD-L1+^@^	PD-L1-
TIL+*	**Type I**	**Type IV**
	13.6% (58/428), Mean OS=30.8 mo	3.3% (14/428), Mean OS=27.0 mo
**TIL-**	**Type III**	**Type II**
	66.1% (283/428), Mean OS=18.3 mo	17.1%(73/428), Mean OS=24.1 mo

^*^ TIL+ was defined as high TIL density (score of 3)

^@^PDL1+ was defined as showing ≥1% PD-L1 expression on tumor cell membranes with ≥1+ intensity.

## DISCUSSION

In the present study, we investigated tumor PD-L1 expression as an independent prognostic marker in ESCC patients. We also examined the importance of TILs in the tumor microenvironment. Early reports examined PD-L1 expression in smaller ESCC populations [19, 20], and results in our study are consistent with these previous reports [19, 20]. Analysis of the correlation between PD-L1 expression and clinicopathologic features suggests that tumor PD-L1 positivity might be associated with more aggressive tumor progression, including deeper tumor invasion and more nodal metastasis, which significantly negatively affects the survival of ESCC. Indeed, in our study, patients with PD-L1+ tumors were more likely to have worse outcomes, including those who underwent surgery or (chemo-)radiation. To explain this phenomenon, we must focus on the function of PD-1/PD-L1 pathway, which conveys an inhibitory signal to T cells and thus impedes immune responses [21, 22]. Multiple solid tumor types, including melanoma [23], RCC [24, 25], NSCLC [14, 26], bladder [27], breast [28], and hepatocellular carcinoma [29], generate an immunosuppressive tumor microenvironment by expressing PD-L1, thereby avoiding T cell-mediated cytolysis. Therefore, tumor PD-L1 overexpression confers a worse prognosis across multiple tumor histologies [30]. Similar to previous studies [27, 31, 32], we choose a 1% PD-L1 cut-off in this study, and found that PD-L1+ patients who received curative esophagectomy or definitive chemo-(radiation) therapy had significantly shorter DFS and OS than PD-L1– patients, and these results provide a rationale for therapeutic intervention targeted to this immunomodulatory axis in these two groups, such as adjuvant therapy with an anti-PD-1/anti-PD-L1 antibody or concurrent anti-PD-1/anti-PD-L1 antibody and radiation treatment [33]. Neither PFS nor OS were significantly different between PD-L1+ and PD-L1– patients who received palliative chemotherapy, which might because of the wide variety of chemotherapy regimens and cycles. Two studies recently defined the tumor cell PD-L1 expression score by membrane and cytoplasmic staining or cytoplasmic staining alone [34, 35]. In this study, we mainly used membranous staining as a basis for staining, but also attempted to assess PD-L1 expression in specimens using stromal cells and immune cells. To our disappointment, no difference was found using these two criteria. This suggests that PD-L1 expression on tumor cell membranes plays a more important role in the prognosis of ESCC patients.

Presence or absence of TILs is a very important factor which should be taken into account when considering administering use of an immune checkpoint inhibitor, since link to efficacy of immune checkpoint blockade requires immune cell infiltration of the tumors [14, 23, 36]. A high degree of CD8+ and CD4+ T-cell infiltration in ESCC correlated with favorable clinical outcome, suggesting that tumor antigen-specific cellular immune response plays a role in the clinical course of the disease [16, 17]. In colorectal cancer, patients with microsatellite unstable (MSI-H) primary tumors have a superior prognosis, associated with increased TILs within tumors [36, 37] and due to MSI-H tumors having an increased mutational burden resulting in increased neoantigen formation. In this study, we qualitatively evaluated TILs and found that PD-L1 expression was positively associated with TIL density. An earlier study by Taube et al. also demonstrated that the presence of TILs was strongly associated with local tumor PD-L1 expression in melanoma patients [38]. One potential explanation for this result may be that inflammatory cytokines, particularly interferon-gamma, released by TILs would induce upregulation of PD-L1 in tumors as an adaptive immune-resistance mechanism to suppress local effector T-cell function against autoimmune attack [39]. In addition, we also found that high TILs were associated with a longer OS in patients who underwent surgery. We classified ESCC tumors into four different types based on the presence or absence of TILs and PD-L1 expression in the microenvironment based on the study reported recently [40]. Incidence of a type I (PD-L1+ and & TIL+) tumor microenvironment was found in 13.6% of ESCC specimens, which was lower than that found in advanced melanoma (38.0%) [15, 38], and this is the group believed to be representis largely responding to checkpoint blockade because of preexisting intratumoral T cells whose activity is attenuated by PD-L1 engagement. The dominant type in ESCC was type III, which was defined as PD-L1+ and & TIL–; for patients of this type it may be worthwhile to consider approaches to promoted T cell infiltration of tumors. For example, radiotherapy could induce immunogenic cell death to liberate neoantigens and promote T-cell infiltration of the tumor. Concurrent use of anti-PD1/PD-L1 therapy may improve effect [41]. The proportions of patients with type II (PD-L1– and TIL–) and type IV (PD-L1– and TIL+) ESCC were very small (17.1% and 3.3%) and were lower than found in melanoma (approximately 41% and 20%). For type II patients, single-agent checkpoint blockade would not be expected to be effective, due to the lack of PD-L1 expression and pre-existing T cell infiltration [40]. Combination therapy designed to promote T cell infiltration but avoid negative regulation mitigate their attenuation, such as the combination of anti–CTLA-4 and anti–PD-1, might lead to a more robust response [42, 43]. For type IV cancers, TIL function might be targeted by other non–PD-1/PD-L1 checkpoint receptors. This types of therapeutic approaches are mostly still in their infancy and more mature data is required.

We also collected 17 paired specimens, 9 collected before and after 2 cycles of chemotherapy, 5 collected before and after 4 or more cycles of chemotherapy, and 3 collected in patients who did not receive any treatment between harvesting tumor samples (when these patients first visited the hospital, endoscopic samples were collected for diagnosis but patients refused treatment due to personal reasons; after one month or longer, patients who visited the hospital again and had a second sample collected to evaluate the patient's condition). As a validation for our hypothesis that PD-L1 expression is associated with an aggressive tumor progression, we found that PD-L1 expression increased as disease progressed. In contrast, PD-L1 expression decreased between baseline and second tumor sampling obtained after 2 cycles of chemotherapy in those with satisfactory treatment responses. Interestingly, PD-L1 expression increased after 4 or more cycles of chemotherapy in patients with worse treatment responses. Recently, a study investigated changes of PD-L1 after neoadjuvant therapy in ESCC patients. Similarly, they found a decrease in PD-L1 expression after 2 cycles of neoadjuvant chemotherapy (NAC) and an increase after neoadjuvant concurrent chemoradiotherapy [35]. However, the authors compared PD-L1 expression between diagnostic endoscopic biopsies and surgical specimens, and it is not clear whether the size of samples would affect results in his study. In our study, PD-L1 expression increased or decreased according to chemotherapy response. To date, there is no confirmed explanation for this phenomenon. We speculate that chemotherapy results in tumor shrinkage resulting in proportionally PD-L1 expression decreases, but when chemotherapy is no longer effective, tumor regrowth lead to an increase in PD-L1 expression. This might suggest that anti-PD-1/PD-L1 therapy as last-line therapy may be effective for heavily pre-treated ESCC patients [44]. However given our sample size was small, with only 17 pairs, further studies with large sample sizes are needed to confirm our data.

We also investigated the immunological impact of chemotherapy on CD8+ and Foxp3+ T cells in the ESCC tumor microenvironment. Among 14 patients who received chemotherapy, 10 exhibited an increase in CD8+ T cells. Increased levels of CD8+ T cells were associated with a better outcome, although the difference was not significant due to small number of available samples. This finding was also seen in several other studies [14, 45–47], including a study in NSCLC in which CD8 was found to be an independent prognostic factor [45]. We also found that increased infiltration of CD8+ T lymphocytes into the tumor microenvironment was associated with a better response to chemotherapy. Considering the results, the cytotoxic effects of chemotherapy could result in release of various tumor antigens being into the local microenvironment, which would then inducing infiltration of T lymphocytes such as CD8+ T cells exert their antitumor activity [14]. A proportion of the residual tumor cells will still express PD-L1, combination of chemotherapy with anti-PD1/PD-L1 might enhance antitumor activity. In contrast, no obvious changes or associations were observed in Foxp3+ T cells populations.

In conclusion, PD-L1 is expressed on the cells of most (>70%) ESCC tumors, and is significantly associated with tumor aggressiveness and an enhanced risk for postoperative recurrence (shorter DFS and OS). As such, PD-L1 may represent a target for treatment of ESCC and is a potentially predictive biomarker for anti-PD-1/PD-L1 antibodies. The results presented in this study support the clinical development of PD-1/PD-L1 blockade as a treatment for ESCC. Further research investigating the effect of PD-L1/PD-1 blockade in ESCC is warranted.

## MATERIALS AND METHODS

### Tissue collection

Archival formalin-fixed, paraffin-embedded ESCC samples were collected from patients diagnosed with primary ESCC after surgery or by endoscopic biopsy at the Affiliated 307 Hospital Cancer Center, Beijing, P. R. China between 2004 and 2014. Serial sections of 4 μm thickness were obtained from each specimen. Histopathologic data was obtained from pathology reports, and patient survival data was obtained from the patient's attending physician. The retrieval of tissue and clinical data were performed according to the regulations of the local institutional review boards and data safety laws with specific regard to ethical standards and patient confidentiality (Ethics No. ky-2016-2-12).

### Patient information and treatment

Conventional clinicopathologic variables, including age, gender, level of differentiation, primary tumor location, tumor invasion depth (T), nodal metastasis (N), distant metastasis (M), TNM stage, and treatment received were collected from patients' attending physicians. Tumor stage was determined according to the 7th American Joint Committee on Cancer/International Union Against Cancer tumor-node-metastasis (TNM) classification system (2009).

A subset of patients underwent right or left thoracotomy for curative resection by total or subtotal thoracic esophagectomy, in addition to regional lymphadenectomy; none underwent transhiatal esophagectomy. Regional lymph nodes included not only mediastinal but also perigastric nodes, thus regional lymphadenectomy represented at least a two-field lymphadenectomy. Esophageal reconstruction was performed using the stomach, colon, or jejunum. Postoperative treatment was recommended for patients with pathologically confirmed metastasis to the lymph nodes or deep tumor invasion (≥T3), and patients without severe postoperative complications. Postoperative treatment included adjuvant chemotherapy, radiotherapy or chemoradiotherapy. The first cycle of adjuvant chemotherapy was administered 4-5 weeks after surgery, and consisted of cisplatin and 5-FU or cisplatin and paclitaxel at standard doses. Adjuvant radiation therapy was administrated 4-5 weeks after surgery, and included external beam radiation with four fields of 6 MV photons to a total dose of 50.4-60 Gy using 1.8-2 Gy daily fractions in 5 weeks. Positioning of the fields and dosimetry were studied using a CT scan and 3D treatment plan. For adjuvant chemoradiotherapy, the radiation therapy program was the same as that described above, and chemotherapy regimens consisted of cisplatin or paclitaxel or 5-FU administered concurrently with radiation.

Patients who were unfit for surgery due to the presence of comorbidities or other risk factors received definitive (chemo-)radiation at ≥50.4 Gy. For tumors of the cervical esophagus, high dose (60-66 Gy) definitive (chemo)-radiation therapy was prescribed. Patients with metastatic or unresectable esophageal cancer received palliative chemotherapy. Chemotherapy regimens consisted of cisplatin, taxanes (paclitaxel or docetaxel), fluorouracil (5-FU, capecitabin, S-1) or others. All chemotherapeutic drugs were prescribed at standard doses. The clinical criteria for response were defined by RECIST 1.1.

### Immunohistochemistry

Expression of PD-L1 in ESCC tumor specimens was assessed by IHC with an Autostainer Plus (Dako-Agilent Technologies), utilizing a proprietary PD-L1 antibody (rabbit monoclonal antibody clone 73-10, Merck KGaA, Darmstadt, Germany) at 1:1000 dilution. Human tonsil and muscle tissue served as positive and negative control tissue, respectively. Both CD8 and Foxp3 antibodies were purchased from Abcam and used at 1:200 dilution. Sections were baked for 60 min at 60°C in a dehydration oven and dewaxed and rehydrated using xylene and graded alcohol washes. Antigen retrieval and deparaffinization were carried out using the EnVision^TM^ FLEX Target Retrieval Solutions (Dako), and then cooled to room temperature in TBST wash buffer for 5 min, then incubated with the primary antibodies. Antibody detection was visualized using DAB chromagen according to the manufacturer's instructions (Dako). Slides were counterstained with hematoxylin.

### Quantification of PD-L1 expression and immune-cell infiltration

PD-L1 expression on the membranes of tumor cells and TIL/histiocytes was scored blindly. Frequency of PD-L1+ cells and staining intensity were analyzed and every tumor specimen was scored according to the intensity of PD-L1 staining (no staining = 0, weak staining = 1, moderate staining = 2, strong staining = 3). In tumor cells, PD-L1+ by a 1% cut off was defined as specimen with ≥1% membranous expression on tumor cells with ≥1+ intensity, PD-L1+ by a 5% cut off was defined as specimens with ≥5% membranous expression on tumor cells with ≥1+ intensity, and PD-L1+ by a 25% cut off was defined as specimens with ≥25% membranous expression on tumor cells with ≥2+ intensity. Positive PD-L1 expression on lymphoid cells and stromal cells was defined as ≥1% positive cells with membranous staining at ≥1+ intensity. The degree of infiltration by TILs was evaluated from the H&E-stained specimens and scored from 0 to 3: 0 = none; 1 = rare; 2 = moderate and focal infiltration; and 3 = prominent and diffuse infiltration (high). Quantitative evaluation of CD8+ and Foxp3+ cells was carried out using 5 to 10 areas with the most abundant distribution within the tumor using an ocular grid at X200 magnification in a blinded manner. The average numbers of cells were used for statistical analysis.

### Statistical analysis

Disease-free survival (DFS) time was defined as the period from the date of surgery to confirmed tumor relapse date for relapsed patients or from the date of surgery to the date of last follow-up for nonrecurrent patients. Progression-free survival (PFS) time was defined as the period from the date of initiation of first-line chemotherapy to the date of tumor progress or last follow up for patients without tumor progression. All patients who did not experience the event of interest were censored at the last follow-up date.

The distributions of patient and clinical characteristics between PD-L1+ and PD-L1- were compared using Chi-square test, Wilcoxon rank-sum test, or two-sample T test, when appropriate. The correlation between PD-L1 expression and other factors (e.g. TILs, stage) was analyzed using Spearman's rank correlation. Survival curves were estimated by the Kaplan-Meier method and compared using the log-rank test. The stepwise Cox-regression model was used to identify factors significantly related to OS and DFS (PFS). P<0.05 (two-tailed) was considered to indicate statistical significance. All statistical analyses were performed using SAS 9.3.

## SUPPLEMENTARY FIGURES


