# Supplementary material for: Prognostic significance of tumor-infiltrating immune cells and PD-L1 expression in esophageal squamous cell carcinoma
Source: Oncotarget. 2017 Feb 22;8(18):30175–89. doi: 10.18632/oncotarget.15621 (PMC5444735; doi:10.18632/oncotarget.15621)
Supplement: Supplementary file 1 [file oncotarget-08-30175-s001.pdf]

Prognostic significance of tumor-infiltrating immune cells and PD-L1 expression in esophageal squamous cell carcinoma

SUPPLEMENTARY FIGURES

A

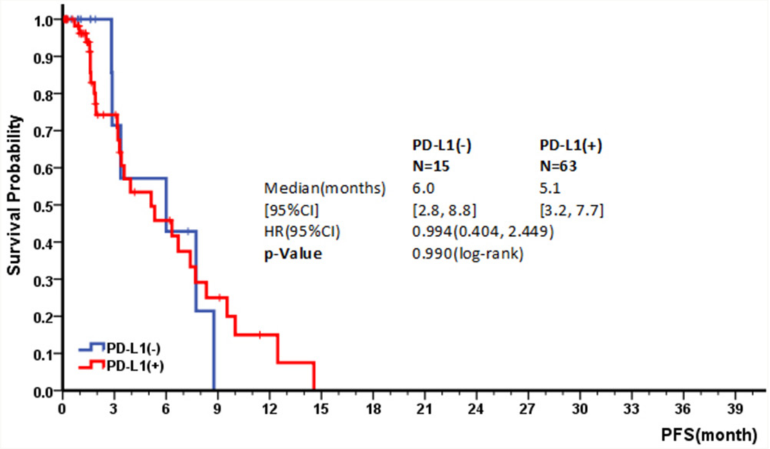

B

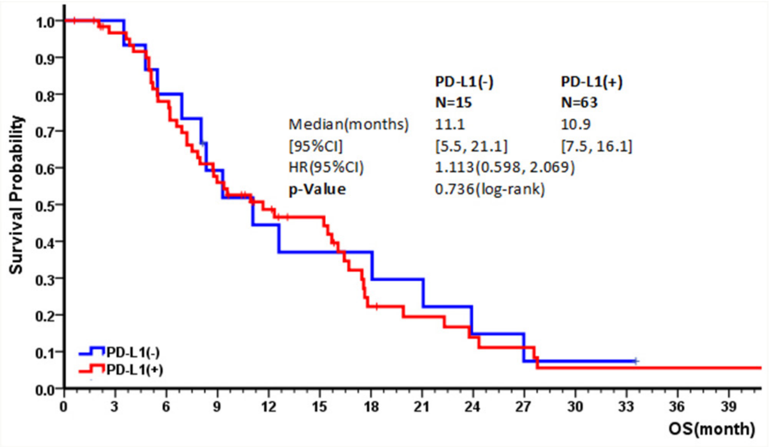

Supplementary Figure 1: Kaplan–Meier survival curves for PFS and OS by PD-L1 expression. A & B: Correlation of PD-L1 expression and PFS/OS in the palliative chemotherapy cohort.

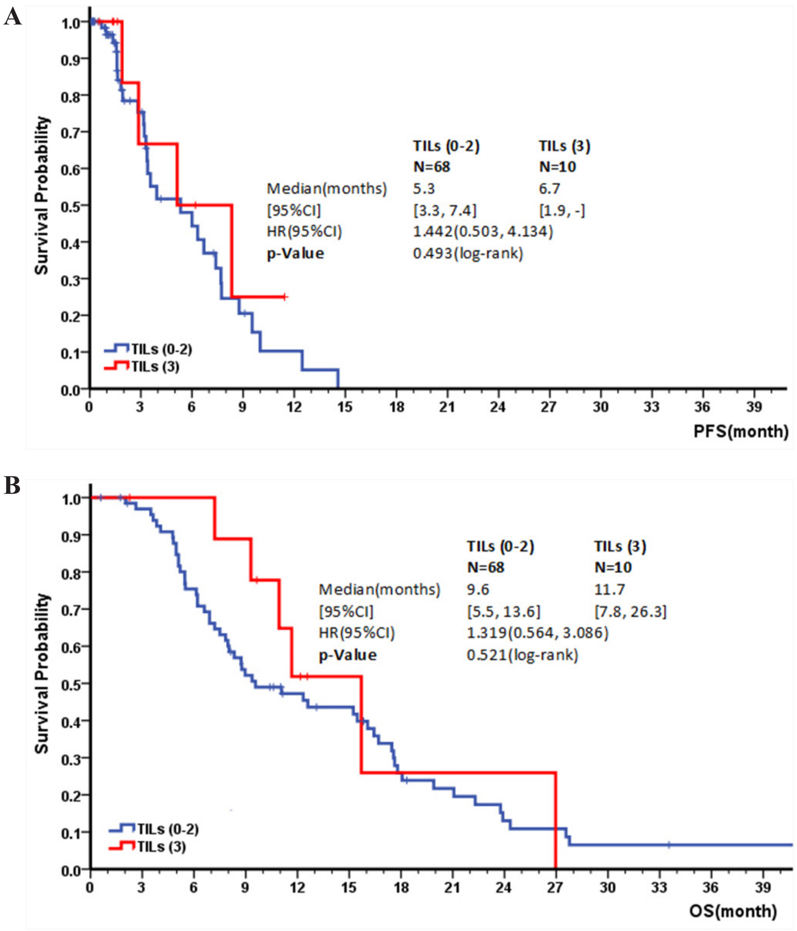

Supplementary Figure 2: Kaplan–Meier survival curve for PFS and OS depending on TIL level: A&B: Correlation of TIL level and PFS/OS in the palliative chemotherapy cohort.
